# Intranasal Immunization with Influenza Virus-Like Particles Containing Membrane-Anchored Cholera Toxin B or Ricin Toxin B Enhances Adaptive Immune Responses and Protection against an Antigenically Distinct Virus

**DOI:** 10.3390/v8040115

**Published:** 2016-04-21

**Authors:** Xianliang Ji, Zhiguang Ren, Na Xu, Lingnan Meng, Zhijun Yu, Na Feng, Xiaoyu Sang, Shengnan Li, Yuanguo Li, Tiecheng Wang, Yongkun Zhao, Hualei Wang, Xuexing Zheng, Hongli Jin, Nan Li, Songtao Yang, Jinshan Cao, Wensen Liu, Yuwei Gao, Xianzhu Xia

**Affiliations:** 1 College of Veterinary Medicine, Inner Mongolia Agricultural University, Huhhot 010018, China; jixianliang2011@126.com (X.J.); jinshancao@imau.edu.cn (J.C.); 2 Institute of Military Veterinary, Academy of Military Medical Sciences, Changchun 130122, China; renzhiguang66@126.com (Z.R.); meng13843191452@163.com (L.M.); zhijun0215@gmail.com (Z.Y.); fengna0308@126.com (N.F.); xysang2012@163.com (X.S.); 18626656520@126.com (S.L.); liyuanguo0520@163.com (Y.L.); wgcha@163.com (T.W.); zhaoyongkun1976@126.com (Y.Z.); wangh25@hotmail.com (H.W.); zhengxx2513@163.com (X.Z.); jin8616771@163.com (H.J.); linan226@126.com (N.L.); yst62041@163.com (S.Y.); 3 Institute of Laboratory Animal Sciences, Chinese Academy of Medical Sciences Peking Union Medical College, Beijing 100730, China; 4 Key Lab of Cellular and Molecular Immunology, Henan University School of Medicine, Kaifeng 475001, China; 5 Jilin Medical University, Changchun 132013, China; doudou7756@126.com; 6 College of Animal Science and Technology, Jilin Agricultural University, Changchun 130118, China; 7 Jiangsu Co-innovation Center for Prevention and Control of Important Animal Infectious Diseases and Zoonoses, Nanjing 210009, China; 8 School of Public Health, Shandong University, Jinan 250110, China

**Keywords:** influenza, virus-like particles, membrane-anchored cholera toxin B, ricin toxin B, cross-protection, intranasal administration

## Abstract

Vaccination is the most effective means to prevent influenza virus infection, although current approaches are associated with suboptimal efficacy. Here, we generated virus-like particles (VLPs) composed of the hemagglutinin (HA), neuraminidase (NA) and matrix protein (M1) of A/Changchun/01/2009 (H1N1) with or without either membrane-anchored cholera toxin B (CTB) or ricin toxin B (RTB) as molecular adjuvants. The intranasal immunization of mice with VLPs containing membrane-anchored CTB or RTB elicited stronger humoral and cellular immune responses when compared to mice immunized with VLPs alone. Administration of VLPs containing CTB or RTB significantly enhanced virus-specific systemic and mucosal antibody responses, hemagglutination inhibiting antibody titers, virus neutralizing antibody titers, and the frequency of virus-specific IFN-γ and IL-4 secreting splenocytes. VLPs with and without CTB or RTB conferred complete protection against lethal challenge with a mouse-adapted homologous virus. When challenged with an antigenically distinct H1N1 virus, all mice immunized with VLPs containing CTB or RTB survived whereas mice immunized with VLPs alone showed only partial protection (80% survival). Our results suggest that membrane-anchored CTB and RTB possess strong adjuvant properties when incorporated into an intranasally-delivered influenza VLP vaccine. Chimeric influenza VLPs containing CTB or RTB may represent promising vaccine candidates for improved immunological protection against homologous and antigenically distinct influenza viruses.

## 1. Introduction

Seasonal influenza A viruses are human respiratory pathogens that cause seasonal epidemics and an average of 250,000–500,000 annual deaths globally [1]. Newly emergent influenza viruses are a persistent public health threat, as underscored by the novel 2009 H1N1 pandemic virus and sporadic human cases of avian H5N1 and H7N9 influenza viruses [2]. Vaccination remains the most effective approach to prevent and control both seasonal and pandemic influenza.

Humans are highly susceptible to newly emergent pandemic influenza viruses due to a lack of pre-existing immunity. Currently available influenza vaccines are limited in their capacity to elicit highly cross-reactive immune responses against antigenically novel viruses [3]. Moreover, the sporadic nature of the pandemic influenza virus emergence poses a logistical problem for vaccine manufacturing due to the time it takes to produce and distribute adequate doses of an effective vaccine. Thus, there is a need to develop influenza vaccine formulations that are capable of eliciting protective immunity against antigenically diverse influenza viruses at low doses.

Virus-like particles (VLPs) are self-assembling structures composed of one or more viral proteins. While the size and structure of VLPs closely resemble those of native viruses, VLPs do not contain viral genetic material and are therefore non-infectious and do not require biosafety containment [4]. Importantly, VLPs produced using baculovirus-based expression systems have the capacity for industrial-scale yields and reduced manufacturing times [5]. VLPs can be constructed of immunologically relevant viral antigens that elicit both innate and adaptive immune responses and may therefore be used as vaccine agents [6]. VLPs have been generated and tested as vaccine candidates for several viruses [7,8,9,10,11]. Influenza VLPs have been reported to induce high neutralizing antibody titers and strong protective immunity [12,13,14,15,16,17]. However, while a single immunization with influenza VLPs can protect mice against homologous viruses, they failed to induce complete protection against antigenically distinct viruses [16,18].

The incorporation of membrane-anchored molecular adjuvants may enhance the immunogenicity of VLP-based vaccines. Cholera toxin B (CTB) is a powerful mucosal adjuvant that enhances mucosal antibody responses and protective immunity during vaccination. CTB is a non-toxic component of cholera toxin (CT) that binds to GM1 gangliosides commonly expressed on the plasma membrane of all eukaryotic cells [19]. CTB has been reported to be safe to administer to humans [20]. When delivered mucosally, CTB can act as an efficient carrier molecule for linked antigens [21]. Importantly, CTB stimulates the upregulation of costimulatory molecules on antigen-presenting cells (APCs), leading to enhanced induction of antigen-specific T and B cell responses [22,23,24]. CTB has also been shown to enhance the immune response to mucosally administered antigens [25]. A single dose of an intranasal influenza HA vaccine containing CTB induced high levels of virus-specific IgA and hemagglutination-inhibiting antibodies that protected animals against viral challenge [26].

The ricin toxin B subunit (RTB) has also been applied as an adjuvant for mucosally-delivered vaccines [27,28]. The terminal galactose of the nontoxic RTB carbohydrate-binding subunit directs binding to glycolipids and glycoproteins present on almost every cell type [29], and it has a broader receptor-binding specificity than CTB or *E. coli* heat labile toxin B [30]. Like CTB, RTB can serve as a carrier to facilitate the uptake of linked antigens and enhance subsequent mucosal immune responses [27].

Intranasal administration of vaccines represents an important approach to induce mucosal immune responses that provide a first line of defence against mucosally acquired pathogens like influenza [31]. This procedure is simple, reliable, and inexpensive compared with other routes of administration [32]. In the present study, we developed chimeric VLPs (cVLPs) containing the HA, NA and M1 proteins of A/Changchun/01/2009 (H1N1) and the membrane-anchored form of either CTB or RTB. The ability of cVLPs to enhance adaptive immune responses and confer protection against a high-dose virus challenge following vaccination was assessed.

## 2. Materials and Methods

### 2.1. Cell Lines and Viruses

Spodoptera frugiperda (Sf9) insect cells (Invitrogen, Carlsbad, CA, USA) were cultured in TMN insect medium (AppliChem, Damstadt, Germany) at 27 °C with shaking at 120 rpm. Madin-Darby canine kidney (MDCK) cells were cultured and maintained in Dulbecco’s modified Eagle’s medium (DMEM) containing 10% fetal bovine serum.

A/Changchun/01/2009 (H1N1) was isolated and stored at the Changchun Veterinary Research Institute. Mouse-adapted UI182 virus was generated by serial passage of A/Changchun/01/2009 in mice and showed similar antigenicity to A/Changchun/01/2009 virus, as previously described [33]. Mouse-adapted FM1-6 virus was derived from A/Fort Monmouth/1/1947(H1N1) and was kindly provided by the Chinese Center for Disease Control and Prevention. The amino acid sequence homology between the UI182 virus and FM1-6 virus is 74.3% in the HA (1701 bp) subunit. The NA (1410 bp) and M1 (759 bp) amino acid sequence homologies of the UI182 virus were 83.6% and 93.7%, respectively, compared to the FM1-6 virus. Viruses were grown in 10-day-old embryonated chicken eggs and purified from allantoic fluid using a discontinuous sucrose gradient of 20%, 30% and 60%. Purified virus was mixed with formalin at a final concentration of 1:4000 (*vol/vol*) as described previously to inactivate the virus [33]. Mice were infected with serial dilutions of mouse-adapted UI182 and FM1-6 viruses, and the 50% lethal dose (LD_50_) was determined.

### 2.2. Construction of Membrane-Anchored RTB or CTB Coding Sequences

Constructs expressing membrane-anchored forms of RTB and CTB were produced via chemical synthesis by Shanghai Generay Biotech (Shanghai, China) as follows. The coding sequence of the honeybee melittin signal peptide (SP) was appended to the 5′ end of the full-length coding sequence of CTB and RTB (GenBank accession no. HQ224500.1 and E01356.1, respectively). The transmembrane (TM) and cytoplasmic tail (CT) coding sequences of the hemagglutinin (HA) gene of A/Changchun/01/2009 (H1N1) were appended in-frame to the 3′ end of the RTB and CTB coding sequences [34]. Sequences encoding membrane-anchored RTB and CTB were cloned into the pFastBac1 vector under the control of the polyhedron promoter (Invitrogen) following EcoRI and NotI digestion.

### 2.3. Generation of Recombinant Baculoviruses and Production of cVLPs

A/Changchun/01/2009 (H1N1) viral RNA was extracted using the High Pure Viral RNA Kit (BioFlux, Tokoyo, Japan). The HA, NA, and M1 coding sequences were amplified from extracted RNA by a reverse transcription-polymerase chain reaction (RT-PCR) using gene-specific oligonucleotide primers containing either EcoRI or NotI restriction sites (Table 1). The PCR products were separately cloned into the pFastBac1 vector (Invitrogen) following the digestion of them and the plasmid with EcoRI and NotI. Recombinant baculoviruses (rBVs) expressing membrane-anchored RTB or CTB and the HA, NA, and M1 proteins from the A/Changchun/01/2009(H1N1) virus were generated using the Cellfection^®^II Reagent (Life Technologies, San Diego, CA, USA) in Sf9 insect cells according to the manufacturer’s instructions. Influenza VLP was produced by co-infecting with rBVs expressing HA, NA and M1 in Sf9 insect cells. To produce chimeric VLPs containing A/Changchun/01/2009(H1N1) virus HA, NA and M1 and RTB or CTB, the Sf9 insect cells were co-infected with rBVs expressing these proteins. Culture supernatants containing VLPs were harvested at day 5 post-infection, and cellular debris was removed by low-speed centrifugation (2000 rpm for 20 min at 4 °C). VLPs were pelleted by ultracentrifugation at 30,000 rpm for 60 min and then purified through a 20%–30%–60% discontinuous sucrose gradient at 30,000 rpm for 1 h at 4 °C.

### 2.4. Immunofluorescence Assay (IFA)

Sf9 cells were separately infected with each rBV to characterize protein expression by IFA. Following infection, the cells were cultured at 27 °C in 5% CO_2_ for 48 h, as previously described [35]. After discarding the supernatant, the cells were fixed with 80% pre-cooled acetone at −20 °C for 2 h. The cells were washed in phosphate-buffered saline (PBS) and incubated with H1N1-reactive chicken antisera (kindly provided by Yu Zhijun) at a dilution of 1:500 specific for HA, NA, or M1 or mouse anti-CTB antibody (Sigma, St. Louis, USA) or mouse anti-RTB antibody (kindly provided by Liu Wensen) at a dilution of 1:500 at 37 °C for 2 h. Finally, the cells were stained with FITC-conjugated rabbit anti-chicken or goat anti-mouse antibodies (Bioss, Beijing, China) diluted in 0.1% Evans blue at 37 °C for 1 h. Cells were analysed under a fluorescence microscope.

### 2.5. Western Blot Analysis

Evaluation of VLP protein content was assessed by western blot. Purified VLPs were separated by sodium dodecyl sulfate (SDS) polyacrylamide gel electrophoresis using a 12% polyacrylamide gel, as previously described [36]. The samples were transferred onto a PVDF membrane using a Mini Trans-Blot system (Bio-Rad, Berkeley, CA, USA). For HA, NA and M1 expression analysis, membranes were probed with an H1N1-reactive chicken antiserum (kindly provided by Yu Zhijun) at a dilution of 1:500 followed by incubation with a horseradish peroxidase (HRP)-conjugated goat anti-chicken IgG secondary antibody (1:50,000 *v*/*v*, Sigma). For analysis of CTB and RTB expression, membranes were probed with mouse anti-CTB or mouse anti-RTB antibodies at a dilution of 1:1000 overnight at 4 °C, followed by an HRP-conjugated goat anti-mouse secondary antibody (Sigma) at a dilution of 1:50,000 for 60 min at 37 °C. Antibody binding was visualized using the ECL system (Thermo, Waltham, MA, USA).

### 2.6. Electron Microscopy

cVLPs were applied to a carbon-coated formvar grid and stained with 1% phosphotungstic acid for 1–2 min. Excess stain was removed with filter paper, and the samples were air dried for 1–3 min [37]. cVLPs were observed using an H8100 transmission electron microscope (Hitachi, Ltd., Hitachi, Japan) at a magnification of 40,000×.

### 2.7. Immunization and Challenge

Protein concentrations of purified influenza VLPs and cVLPs were analysed using a BCA assay kit (Thermo). Female BALB/c mice aged 6–8 weeks (Changchun Animal Breeding Center for Medical Research, Changchun, China) were immunized intranasally two times at 2-week intervals with 0.2 mL containing 10 µg (2^7^ HA units) VLPs, CTB-VLPs or RTB-VLPs. As a mock immunization control, mice were immunized with PBS alone. Two weeks following the booster immunization (week 5 of immunization schedule), the mice were anesthetized with isoflurane and intranasally infected with 10 MLD_50_ of mouse-adapted UI182 or FM1-6 in 50 µL of PBS. The body weights and survival were monitored daily. Mice losing >25% of their starting body weight were considered moribund and were euthanized according to predetermined endpoints set by the Institutional Animal Care and Use Committee (IACUC). All animal experiments were performed in accordance with the ethical guidelines of the International Association for the Study of Pain and were approved by the Animal Care and Use Committee of the Chinese People’s Liberation Army (No:SYXK2009-045).

### 2.8. Antibody Responses and Hemagglutination-Inhibition (HAI) Titers

Blood samples were collected by retro-orbital plexus puncture immediately prior to the first immunization (week 0), immediately prior to the booster immunization (week 3) and two weeks following the booster immunization (week 5). Blood was transferred to a tube containing a serum separator and clot activator and incubated at room temperature. The tubes were centrifuged, and the sera were removed and frozen at −20 °C prior to antibody titration. Lung and nasal wash samples were collected from immunized mice on day 4 post-challenge with 10 MLD_50_ UI182 or FM1-6 virus. Nasal washes were collected by lavage using 250 µL PBS. Mouse lungs were lavaged repetitively with 1 mL PBS to collect lung lavage. Samples were centrifuged, filtered, and stored at −80 °C, as previously described [38].

Levels of influenza virus-specific antibody in sera, nasal washes, and lung washes were determined by enzyme-linked immunosorbent assay (ELISA), as described previously [13,39]. In brief, the wells of a 96-well plate were coated with inactivated mouse-adapted UI182 or FM1-6 virus at a concentration of 5 µg/mL in coating buffer (0.1 M sodium carbonate, pH 9.5) at 4 °C overnight. Plates were washed once with PBS and then blocked with 0.1% bovine serum albumin (BSA) in PBS at 25 °C for 2 h. Serial dilutions of each serum, nasal wash and lung wash sample were added to wells and incubated for 2 h at 25 °C. After five washes, the plates were incubated with an HRP-conjugated goat anti-mouse secondary antibody specific for IgG, IgG1, IgG2a, or IgA (1:5000 *v/v*, Southern Biotechnology, Birmingham, AL, USA) at 25 °C for 1 h. Following the five washes, the substrate TMB (Sigma) was used to develop the colour. The reaction was stopped by adding 50 µL of 2 M H_2_SO_4_, and the absorbance was measured at 450 nm using a spectrophotometer (Bio-Rad). HAI titers were determined using 0.85% chicken red blood cells and 4 HA units of mouse-adapted UI182 or FM1-6 per well.

### 2.9. Lung Viral Titers and Microneutralization Assay

Lungs were harvested from immunized mice on day 4 post-challenge with 10 MLD_50_ mouse-adapted UI182 or FM1-6 viruses to determine the viral titers. The lungs were homogenized using frosted glass slides. The homogenates were centrifuged at 3000 rpm for 10 min, and the supernatants collected and stored at −80 °C. The lung homogenates were titrated for virus infectivity in eggs from initial dilutions of 1:10. The limit of virus detection was 10^1.2^ EID_50_/mL [40]. Serum-neutralizing antibody titers were determined by microneutralization (MN) assays using MDCK cells, as previously described [41]. Briefly, 2-fold serial dilutions of inactivated serum samples were mixed with 100 TCID_50_ of mouse-adapted UI182 or FM1-6 virus and incubated at 37 °C for 60 min. The serum/virus mixture was overlaid on MDCK monolayers, and the plates were incubated at 37 °C for 1 h in a 5% CO_2_ cell culture incubator. The inverse of the highest serum dilution in which no cytopathic effect was observed was recorded as the neutralizing antibody titer.

### 2.10. Cytokine Assays Using ELISpot

Enzyme-linked immunospot (ELISpot) assays were performed to characterize the frequency of IFN-γ and IL-4-secreting splenocytes in immunized mice on day 4 post-challenge with mouse-adapted UI182 virus, as described previously [42,43]. Splenocytes (1 × 10^6^ cells) were added to tissue culture wells precoated with anti-mouse IFN-γ or IL-4 antibodies (Mabtech AB, Stockholm, Sweden). Cells were restimulated with 0.2 µg per well of inactivated A/Changchun/01/2009 (H1N1). Splenocytes producing IFN-γ or IL-4 were quantified by ELISpot assay according to the manufacturer’s instructions (Mabtech AB) using an ELISpot reader (Multispotreader Spectrum, AID, Strasberg, Germany).

### 2.11. Statistical Analysis

Data were analysed with the Tukey-Kramer post hoc test of ANOVA for multiple comparisons. Significant differences between the experimental groups were established at *P*-values less than 0.01 or 0.05 (*p* < 0.01 or *p* < 0.05).

## 3. Results

### 3.1. Construction and Characterization of rBVs Expressing CTB or RTB

**Figure 1:**
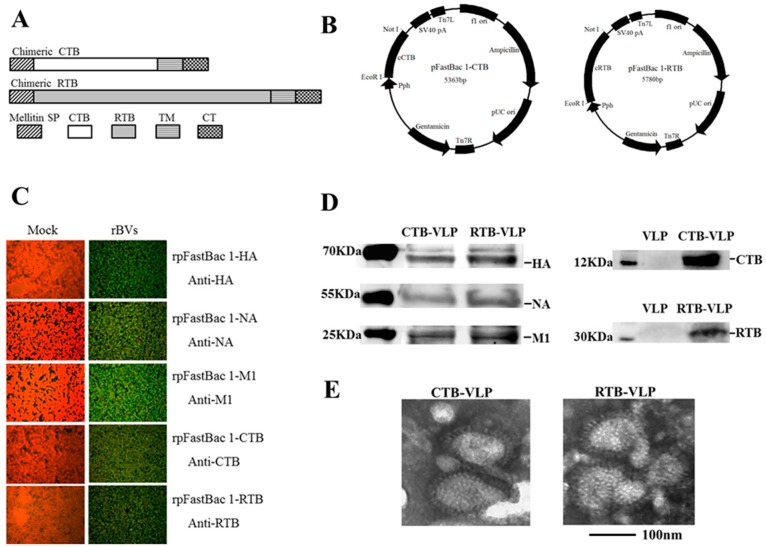
Construction and characterization of cVLPs containing membrane-anchored CTB or RTB. (**A**) Schematic diagrams of membrane-anchored CTB and RTB fusion proteins; (**B**) Schematic diagrams of pFastbac1-CTB and pFastbac1-RTB recombinant plasmids used to generate baculoviruses expressing membrane-anchored CTB or RTB; (**C**) Analysis of HA, NA, M1, CTB, and RTB expression following infection of insect cells with recombinant baculoviruses by indirect immunofluorescence; (**D**) Immunoblot analysis of cVLP protein content; (**E**) Electron microscopy of cVLPs. Scale bar represents 100 nm.

A recombinant baculovirus expression system was used to generate VLPs consisting of the HA, NA, and M1 proteins of A/Changchun/01/2009/ (H1N1) with or without the membrane-anchored versions of CTB or RTB using previously described methods [36]. The membrane-anchored versions of CTB and RTB were constructed by fusing the CTB and RTB coding sequences with sequences encoding the honeybee melittin signal peptide and the transmembrane and cytoplasmic regions of the A/Changchun/01/2009 (H1N1) HA protein (Figure 1A). The sequences encoding chimeric CTB and chimeric RTB were cloned into the pFastBac1 vector to generate the recombinant baculoviral plasmids pFastbac1-CTB and pFasatbac1-RTB (Figure 1B). Similarly, the coding sequences of the A/Changchun/01/2009 HA, NA, and M1 proteins were cloned into the pFastBac1 vector to generate the baculoviral plasmids pFastbac1-HA, pFastbac1-NA, and pFastbac1-M1. The recombinant baculoviruses were rescued using each recombinant plasmid following the transfection of Sf9 insect cells. The infection of Sf9 insect cells with each recovered recombinant baculovirus resulted in the expected pattern of protein expression, as shown by indirect immunofluorescence using antibodies specific for HA, NA, M1, CTB, and RTB (Figure 1C).

### 3.2. Production and Characterization of CTB-VLP or RTB-VLP

The chimeric VLPs were produced and purified following the Materials and Methods. The presence of each protein was confirmed by western blot (Figure 1D). Bands corresponding to HA, NA, M1 (64, 51 and 27 kDa, respectively) and CTB (14 kDa) in CTB-VLP and bands corresponding to HA, NA, M1 (64, 51 and 27 kDa, respectively) and RTB (34 kDa) in RTB-VLP were observed. Electron microscopy revealed spherical and pleomorphic VLPs 80–100 nm in diameter, with surface spikes characteristic of influenza virus HA and NA proteins (Figure 1E). These results demonstrate that VLPs containing HA, NA, M1 and either membrane-anchored CTB or RTB are similar to standard influenza VLPs and influenza virions with respect to size and morphology.

### 3.3. cVLPs Containing CTB or RTB Enhance Systemic Antibody Responses

**Figure 2:**
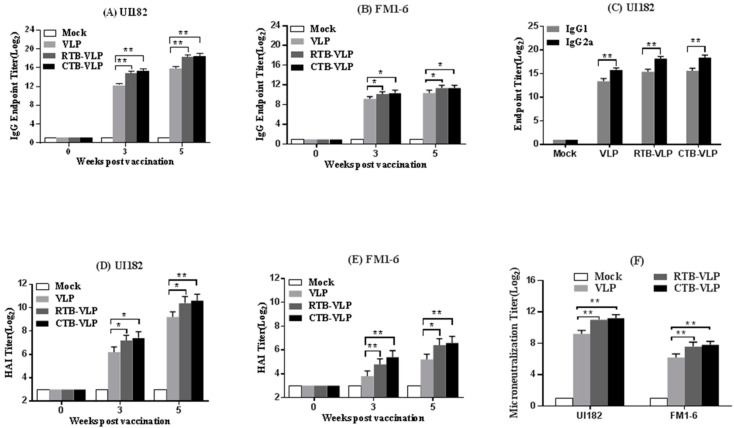
Intranasal immunization with cVLPs enhances systemic antibody responses. (**A**,**B**) Titers of serum IgG specific for UI182 virus (**panel A**) and FM1-6 virus (**panel B**) measured by indirect ELISA; (**C**) Titers of serum IgG1 and IgG2a specific for UI182 virus measured by indirect ELISA; (**D**,**E**) HAI titers against UI182 (**panel D**) and FM1-6 virus (**panel** **E**); (**F**) Virus-neutralizing antibody titers against UI182 and FM1-6 virus were determined for sera collected at week 5 post-vaccination. Data are shown as the means ± SD (n = 5). * *p* < 0.05, ** *p* < 0.01.

Mice were immunized and boosted intranasally with VLPs (containing HA, NA, and M1 proteins), CTB-VLPs (containing HA, NA, M1 and CTB proteins) and RTB-VLPs (containing HA, NA, M1 and RTB proteins) to evaluate the adjuvant effect of membrane-bound CTB and RTB on humoral immune responses. The levels of influenza-specific serum IgG were evaluated by ELISA using mouse-adapted UI182 or FM1-6 viruses 3 and 5 weeks after the primary immunization. The immunization of mice with each VLP preparation elicited detectable influenza-specific serum IgG responses against both UI182 and FM1-6 mouse-adapted viruses (Figure 2A,B). The immunization of mice with CTB-VLPs (3.7 × 10^5^) or RTB-VLPs (3.2 × 10^5^) elicited 5–7-fold higher serum IgG titers against the mouse-adapted UI182 virus when compared with mice immunized with VLPs (5.9 × 10^4^) lacking CTB and RTB at five weeks (Figure 2A, *p* < 0.01). Serum antibody cross-reactivity was tested using the mouse-adapted FM1-6 virus. Mice immunized with CTB-VLPs (2.9 × 10^3^) or RTB-VLPs (2.9 × 10^3^) showed a 2-fold higher IgG titer against FM1-6 virus when compared with mice immunized with VLPs (1.4 × 10^3^) alone, although in general, the antibody titers against FM1-6 were lower than those seen with UI182 (Figure 2B, *p* < 0.05). These results indicate that the incorporation of membrane-anchored CTB or RTB into VLPs enhanced the systemic influenza-specific IgG responses following immunization.

Mice immunized with each VLP preparation developed both influenza-specific IgG1 and IgG2a responses, which are antibody class switch patterns consistent with the generation of both Th1 and Th2 immune response (Figure 2C). Sera from all immunized mice displayed significantly higher levels of IgG2a when compared with IgG1 (*p* < 0.01), irrespective of the specific VLP composition administered, suggesting that mice vaccinated intranasally with VLPs generated a T helper type 1-dominant response. Serum titers of UI182-specific IgG1 and IgG2a were modestly higher in mice immunized with cVLPs when compared with mice immunized with VLPs lacking CTB or RTB (Figure 2C).

We next determined the HAI activity of sera collected 0, 3, and 5 weeks after primary immunization with VLPs, CTB-VLPs, or RTB-VLPs. Immunization with CTB-VLPs (1638) or RTB-VLPs (1434) elicited significantly higher HAI antibody titers against mouse-adapted UI182 virus when compared with titers achieved following immunization with VLPs (614) alone at both 3 and 5 weeks (Figure 2D). HAI antibody titers increased between weeks 3 and 5, irrespective of the VLP vaccine composition (Figure 2D). Further, mice immunized with CTB-VLPs (102) or RTB-VLPs (89) generated significantly higher cross-reactive HAI antibody titers against mouse-adapted FM1-6 virus when compared with titers achieved following immunization with VLPs (38) alone at both 3 and 5 weeks (Figure 2E).

Virus neutralization antibody titers were measured for sera collected from mice immunized with VLPs, CTB-VLPs, or RTB-VLPs 5 weeks following the first immunization. Mice immunized with CTB-VLPs (2457) or RTB-VLPs (2048) had approximately 3-fold higher virus neutralization antibody titers against mouse-adapted UI182 when compared with mice immunized with VLPs (614) alone (Figure 2F, *p* < 0.01). We then evaluated the cross-reactive serum virus-neutralizing activity against FM1-6 virus. While all sera showed higher virus-neutralizing antibody titers against mouse-adapted UI182 when compared with that against the FM1-6 virus, significantly higher FM1-6 virus-neutralizing antibody titers were detected in the sera of mice immunized with CTB-VLP (230) or RTB-VLP (204) than in the sera of mice immunized with VLPs (76) alone (*p* < 0.01).

### 3.4. cVLPs Containing CTB or RTB Enhance Mucosal Immune Responses

**Figure 3:**
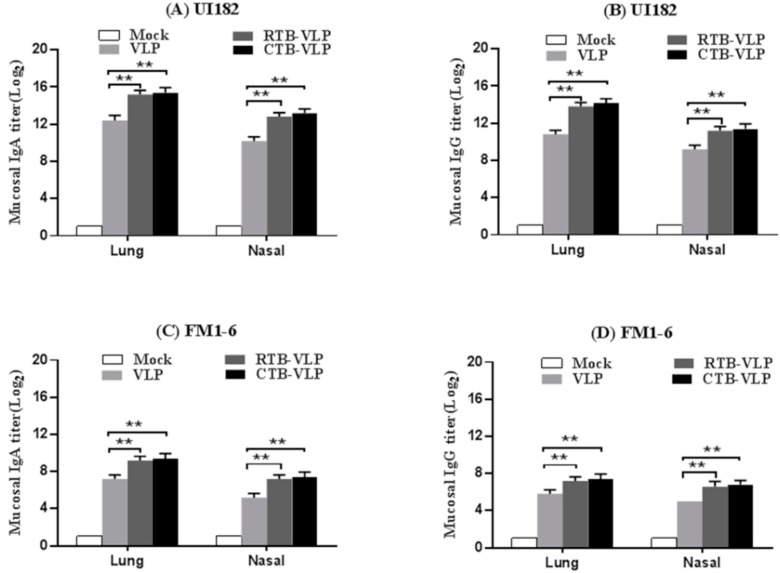
Intranasal immunization with cVLPs enhances mucosal immune responses. (**A**,**B**) Titers of mucosal secretory IgA (**panel A**) and IgG antibodies (**panel B**) specific for UI182 virus in lung and nasal washes collected from immunized mice 4 days post-challenge with 10 MLD_50_ UI182 virus; (**C**,**D**) Titers of mucosal secretory IgA (**panel C**) and IgG antibodies (**panel D**) specific for FM1-6 virus in lung and nasal washes collected from immunized mice 4 days post-challenge with 10 MLD_50_ FM1-6 virus.

Respiratory mucosal surfaces are the natural route of entry and the primary replication site of influenza virus. We therefore performed ELISAs to evaluate the levels of mucosal secretory IgA and IgG antibodies in lung and nasal washes collected from immunized mice 4 days following challenge with 10 MLD_50_ UI182 or FM1-6 virus. Lung and nasal washes collected from mice immunized with CTB-VLPs or RTB-VLPs contained significantly higher titers of UI182 virus-specific IgA and IgG when compared with washes collected from mice immunized with VLPs alone (Figure 3A,B). When the lung and nasal washes were assessed for antibody cross-reactivity against the FM1-6 virus, lower titers were seen when compared with the homologous UI182 virus, though mice vaccinated with CTB-VLPs or RTB-VLPs still exhibited significantly higher IgA and IgG titers when compared with mice vaccinated with VLPs alone (Figure 3C,D). These results demonstrate that immunization with cVLPs containing membrane-anchored RTB or CTB significantly enhance mucosal immune responses.

### 3.5. cVLPs Containing CTB or RTB Enhance Cell-Mediated Immune Responses

**Figure 4:**
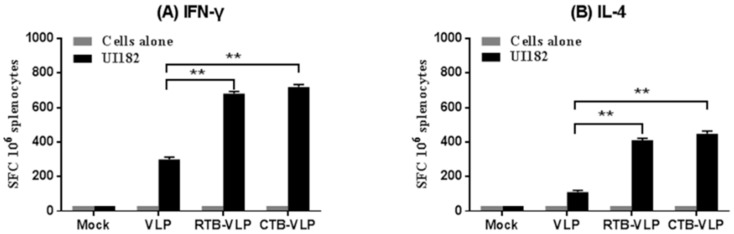
Intranasal immunization with cVLPs increases the frequency of IFN-γ and IL-4-producing splenocytes. (**A**,**B**) Splenocytes were isolated from immunized mice on day 4 post-challenge with 10 MLD_50_ UI182 virus, restimulated with inactivated UI182 virus, and assessed for secretion of IFN-γ (**panel A**) and IL-4 (**panel B**) by ELISpot. Data are shown as the means ± SD (n = 3). * *p* < 0.05, ** *p* < 0.01.

We next evaluated the patterns of T cell cytokine production following vaccination with VLPs, CTB-VLPs, or RTB-VLPs. Splenocytes harvested from immunized mice 4 days post-challenge with 10 MLD_50_ UI182 virus were restimulated with inactivated UI182 virus to enumerate the frequency of IFN-γ and IL-4-secreting cells by ELISpot (Figure 4A,B). Mice immunized with RTB-VLPs or CTB-VLPs had a significantly higher frequency of IFN-γ- and IL-4-secreting cells when compared with mice immunized with VLPs alone (*p* < 0.01), revealing antigen-specific Th1 and Th2 subpopulations of helper T cells. Consistent with the higher titers of virus-specific IgG2a compared to those of IgG1, cells secreting IFN-γ upon restimulation were recovered at a higher frequency than IL-4 secreting cells in all groups, demonstrating a Th1-dominant response. These results suggest that cVLPs containing membrane-anchored RTB or CTB enhance the magnitude of the cellular responses following immunization.

### 3.6. cVLPs Containing CTB or RTB Enhance Heterosubtypic Protection against Lethal Virus Challenge

**Figure 5:**
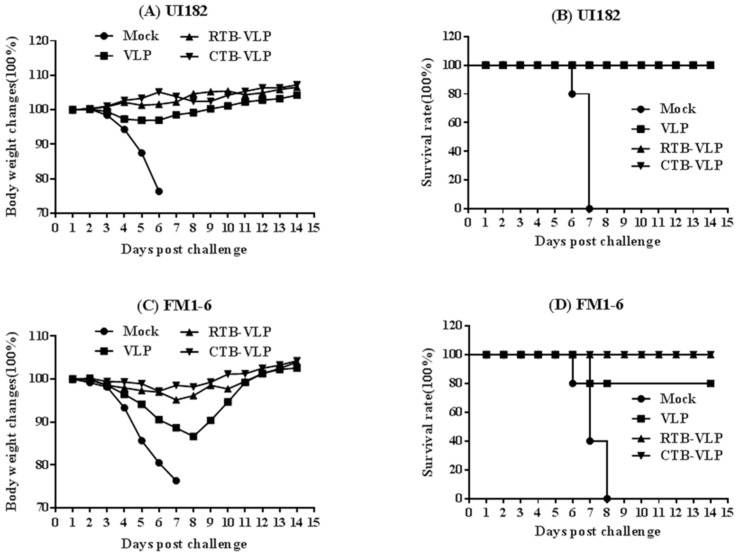
Morbidity and survival in mice immunized with cVLPs following challenge with UI182 or FM1-6 virus. Immunized mice (n = 5) were challenged with 10 MLD_50_ of UI182 (**panels A,B**) or FM1-6 virus (**panels C,D**) 5 weeks after the first immunization. Mice were monitored daily for 14 days for body weight changes (**panels A,C**) and survival (**panels B,D**).

We next assessed whether immunization with VLPs containing membrane-anchored RTB or CTB improved survival following challenge with a lethal dose of UI182 or FM1-6 virus. As expected, mock-immunized mice challenged with 10 MLD_50_ UI182 virus rapidly lost weight and universally succumbed to infection by day 7 post-challenge (Figure 5A,B). In contrast, mice immunized with RTB-VLPs, CTB-VLPs or VLPs alone lost little or no body weight and all survived, indicating the level of immunity conferred by VLPs alone was sufficient to protect mice from a high-dose challenge with homologous virus (Figure 5B).

To evaluate the protective effect of cVLP immunization against heterologous viral challenge, immunized mice were infected with 10 MLD_50_ FM1-6 virus. Mock-immunized mice rapidly lost weight and died by day 8 post-challenge. Mice immunized with VLPs alone lost an average of 15% of their starting body weights and exhibited 20% mortality (Figure 5C,D). In contrast, mice immunized with RTB-VLPs and CTB-VLPs lost only 3% and 5% of their starting body weights, respectively, and universally survived infection with the heterologous FM1-6 virus (Figure 5C,D). These results demonstrate that the increased humoral and cellular immune responses following vaccination with cVLPs containing membrane-anchored RTB or CTB is correlated with improved survival against a lethal dose of heterologous virus when compared with mice vaccinated with VLPs lacking RTB or CTB.

### 3.7. Immunization with cVLPs Containing CTB or RTB Reduces Viral Loads Following Challenge

**Figure 6:**
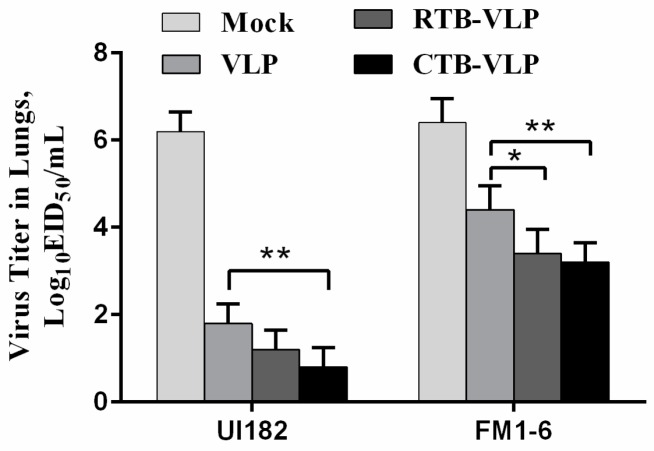
Intranasal immunization with cVLPs results in reduced lung viral loads following lethal challenge. Virus titers in the lungs of immunized mice were measured 4 days post-challenge with 10 MLD_50_ of UI182 virus by titration in eggs. Viral titers are expressed as EID_50_/mL. Data are shown as the means ± SD (n = 3). * *p* < 0.05, ** *p* < 0.01.

As an additional measure of vaccine efficacy, lung viral loads were measured 4 days post-challenge with 10 MLD_50_ homologous UI182 or heterologous FM1-6 viruses. As expected, the mock-immunized mice displayed high titers of both UI182 and FM1-6 in the lungs at 4 days post-challenge relative to mice immunized with VLPs, RTB-VLPs, or CTB-VLPs (Figure 6). The VLP-based immunization of mice resulted in marked reductions in viral loads relative to mock-immunized mice when challenged with either UI182 virus or FM1-6 virus, though the reduction in viral load was more pronounced in immunized mice challenged with the homologous UI182 virus (Figure 6). Mice immunized with VLPs containing membrane-anchored CTB displayed significantly lower lung viral titers of both UI182 and FM1-6 when compared with mice immunized with VLPs alone, whereas immunization with RTB-VLPs resulted in significantly lower viral loads only following challenge with the FM1-6 virus (Figure 6). When challenged with FM1-6 virus, lung viral loads were reduced 10-fold and 15-fold in mice immunized with RTB-VLPs and CTB VLPs, respectively, relative to mice immunized with VLPs alone (Figure 6). These results indicate that cVLPs containing membrane-anchored RTB or CTB resulted in improved viral control following homologous and heterologous virus challenges.

## 4. Discussion

The lack of preexisting immunity to newly emergent pandemic influenza viruses poses a serious public health concern, and the further development of vaccines that elicit protective immunity is needed [16]. A wide variety of VLP-based vaccine candidates have been pursued using different expression systems, some of which have been licensed and commercialized [43]. Ren Zhiguang demonstrated that 10 µg of VLP with or without CFA was sufficient to protect mice from lethal homologous or heterologous challenge and was superior to vaccination with WIV [39]. A VLP vaccine comprising hemagglutinin (HA) and M1 from the A/California/04/2009 virus (H1N1) showed 100% protection against A/PR/8/34 and A/Caledonia/20/99 viruses with only moderate body weight loss and induction of cross-reactive recall and IgG antibody-secreting cell responses [44]. A single intramuscular vaccination with VLPs containing proteins derived from the A/California/04/2009 virus provided complete protection against lethal challenge with the A/California/04/2009 virus and partial protection against the A/PR/8/1934 virus [16]. The incorporation of membrane-anchored molecular adjuvants into VLPs may represent an approach to enhance adaptive immune responses and vaccine efficacy while reducing required antigen doses. Chimeric flagellin-containing influenza VLPs administered via intramuscular or intranasal routes induced high levels of IgA and IgG and enhanced virus-neutralization activity against homo- and hetero-subtypic viruses [34,38]. Similarly, the inclusion of membrane-anchored flagellin and LTB in rabies VLPs improved the survival rates relative to VLPs lacking these adjuvants [43]. Mucosally-delivered vaccines are well-suited to induce protective immunity responses at the primary portals of entry for mucosally-transmitted infectious diseases and induce a broader range of protection against lethal challenge [45,46]. In the present study, we incorporated membrane-anchored CTB or RTB into influenza VLPs and evaluated the immune responses following intranasal immunization. cVLPs induced enhanced humoral and cellular immune responses and provided complete protection against lethal homologous or heterologous influenza virus challenge.

cVLPs containing membrane-anchored RTB or CTB induced higher influenza-specific antibody responses and HAI titers than those induced by VLPs alone. Intranasal immunization with cVLPs was associated with a stronger Th1-biased CD4+ T cell response, as reflected by the increased frequency of IFN-γ-secreting splenocytes upon restimulation. This cellular response was consistent with the observed higher levels of virus-specific IgG2a relative to IgG1, as IFN-γ is recognized to facilitate B cell differentiation to plasma cells and memory cells and drive class-switch recombination to IgG2a [46]. IgG2a is known to play an important role in viral clearance and protection against lethal influenza challenge [47]. These antibodies interact with Fc receptors with high affinity, induce complement activation and stimulate antibody-dependent cellular cytotoxicity [48,49]. Importantly, mice vaccinated with cVLPs showed an increased clearance of virus from the lung upon lethal influenza challenge when compared to mice vaccinated with VLPs alone. The improved humoral response following immunization with cVLPs is likely a major contributor to protection following lethal challenge and effective clearance of virus [16].

cVLPs were generated by the co-infection of Sf9 insect cells with rBVs expressing HA, NA, M1, and either membrane-anchored RTB or CTB. Baculoviruses have densities similar to VLPs and are therefore not efficiently separated from VLPs by density gradient ultracentrifugation [50]. Margine *et al.* reported that residual baculovirus in influenza VLP preparations enhanced immunogenicity and promoted IgG and mucosal IgA responses [51]. Similarly, another report demonstrated that baculoviruses promote dendritic cell maturation and humoral and cytotoxic T cell responses [52]. We detected baculovirus titers in cVLP preparations at approximately 3.4 × 10^5^ pfu/mL. The presence of baculovirus in VLPs may have strong adjuvant properties that further induce strong humoral and cellular immune responses. Safety concerns regarding residual baculoviruses in vaccine preparations for human use still need to be addressed, although a pandemic H1N1 influenza VLP-based vaccine was shown to be both immunogenic and well-tolerated in a double-blind trial after separation from baculoviruses and inactivation of residual baculoviruses [53].

Previous reports have shown that intramuscular vaccination with influenza VLPs effectively protects against lethal challenge with homologous viruses, but only provides partial protection in mice challenged with antigenically distinct heterologous viruses [5,16,46]. In mice intramuscularly vaccinated with an influenza VLP vaccine candidate containing flagellin, 67% of the mice survived the homologous virus challenge infection. Influenza cVLPs incorporating flagellin induced high levels of IgA and IgG secretion and enhanced mucosal virus neutralization activity against homo- or hetero-subtypic viruses compared with standard VLPs and induced higher levels of serum antibody titers with improved neutralization activity and HI titers. Because the HAI titer is known to be correlated with protection in influenza vaccination, these results also support the conclusion that the enhanced antibody responses contribute to the observed cross-protection [34]. Several strategies have been used to confer enhanced protection, including the use of adjuvants. Filippo Ansaldi compared the abilities of MF59^TM^-adjuvanted and non-adjuvanted subunit influenza vaccines containing A/Wyoming/3/03(H3N2) to confer cross-protection against four consecutive drifted strains in the elderly. The MF59^TM^-adjuvanted vaccine induced a stronger booster response against A/Panama/2007/99(H3N2) than the non-adjuvanted vaccine [54]. Two doses of adjuvanted split H5N1 vaccine containing ≥1.7 µg HA induced neutralizing antibodies in the majority of ferrets to both clade 1 (17/23 (74%) responders) and clade 2 viruses (14/23 (61%) responders), and 96% (22/23) of vaccinees survived the lethal challenge. All ferrets in the control groups receiving non-adjuvanted vaccine or adjuvant alone failed to develop specific or cross-reactive neutralizing antibodies, and all died or had to be euthanized within four days of virus challenge [55]. Suryaprakash Sambhara demonstrated that the delivery of H1N1 subtype influenza viral antigens as immunostimulating complexes (ISCOM) induces broad cross-protection in mice against challenge with various influenza virus subtypes, including the avian H9 and the H5 strains that were recently responsible for deaths in humans [56]. The observations of the present study are thus consistent with previous reports. Mice immunized intranasally with VLPs alone via a prime-boost vaccination regimen showed 100% survival following lethal homologous virus challenge. This suggests that the improved immune responses conferred by the inclusion of membrane-anchored CTB or RTB were not necessary for protection at the challenge dose used, though significant reductions in lung viral loads were observed when membrane-anchored CTB or RTB was included in the VLP vaccines. In contrast, when the mice were challenged with a lethal dose of a heterologous virus, mice previously vaccinated with CTB-VLPs or RTB-VLPs showed improved survival relative to mice vaccinated with VLPs alone. While the adjuvant effects of CTB and RTB may not have been necessary to provide immunological protection against homologous virus challenge, the marked enhancement in adaptive immune responses elicited by CTB- and RTB-VLPs improved protection against the antigenically distinct influenza virus. Further investigation is needed to determine the extent of the cross-protective immunity elicited by VLPs incorporating membrane-anchored CTB or RTB against more antigenically distant influenza viruses.

## 5. Conclusions

In conclusion, our results demonstrate that membrane-anchored CTB and RTB possess strong adjuvant activity when incorporated into influenza VLPs. CTB-VLP and RTB-VLP induced higher levels of virus-specific antibodies and cellular immune responses relative to VLPs alone and provided complete protection against lethal homologous or heterologous influenza challenge. VLPs containing membrane-anchored CTB or RTB may represent a safe, convenient and effective mucosal vaccine candidate to prevent influenza infection.

## Figures and Tables

**Table 1:** Sequences of primers used in present study.

Primer	Sequence (5′–3′) *	Restriction Enzyme Site
H1N1 HA F	CCGGAATTCATGAAGGCAATACTAGTAGTTCTGCTATAT	EcoR I
H1N1 HA R	AAATATGCGGCCGCTTAAATACATATTCTACACTGTAGAGACCC	Not I
H1N1 NA F	CCGGAATTCATGAATCCAAACCAAAAGATAATAACCATT	EcoR I
H1N1 NA R	AAATATGCGGCCGCTTACTTGTCAATGGTAAATGGCAACTCAGC	Not I
H1N1 M1 F	CCGGAATTCATGAGTCTTCTAACCGAGGTCGAAACGTAC	EcoR I
H1N1 M1 R	AAATATGCGGCCGCTCACTTGAATCGCTGCATCTGCACTCCCAT	Not I
MSP-CTB-TMCT(HA) F	CCGGAATTCATGAAGTTCCTGGTGAACGTGGCTCTGGTG	EcoR I
MSP-CTB-TMCT(HA) R	AAATATGCGGCCGCTTAGATGCAGATGCGGCACTGCAGGGAACC	Not I
MSP-RTB-TMCT(HA) F	CCGGAATTCATGAAGTTCCTGGTGAACGTCGCCTTGGTC	EcoR I
MSP-RTB-TMCT(HA) R	AAATATGCGGCCGCTTAGATGCAGATACGGCACTGCAAGCTACC	Not I

* Restriction enzyme sites are underlined.
